# Evaluate the accuracy of ChatGPT’s responses to diabetes questions and misconceptions

**DOI:** 10.1186/s12967-023-04354-6

**Published:** 2023-07-26

**Authors:** Chunling Huang, Lijun Chen, Huibin Huang, Qingyan Cai, Ruhai Lin, Xiaohong Wu, Yong Zhuang, Zhengrong Jiang

**Affiliations:** 1 Department of Outpatient Electrocardiography, The Second Affiliated Hospital of Fujian Medical University, Quanzhou, 362000 Fujian China; 2 Department of Endocrinology, The Second Affiliated Hospital of Fujian Medical University, No. 950 Donghai Street, Fengze District, Quanzhou, 362000 Fujian China

**Letter to the editor:**

Epidemiology data reveals a fourfold increase in the global number of diabetes patients over the past three decades, with projections indicating a reach of 642 million by 2040 [1]. More and more patients and their families are relying on the internet for diabetes-related information. In the last 10 years, significant advancements in artificial intelligence have emerged, and ChatGPT has emerged as a prominent AI model. Developed by OpenAI, ChatGPT is part of the Generative Pre-trained Transformer (GPT) model family and was launched on November 30, 2022. By employing deep learning techniques, ChatGPT generates human-like responses to natural language inputs [2]. The primary challenge faced by ChatGPT lies in ensuring the precision and dependability of the system, especially when providing medical advice and information.

## Methods

**Fig. 1:**
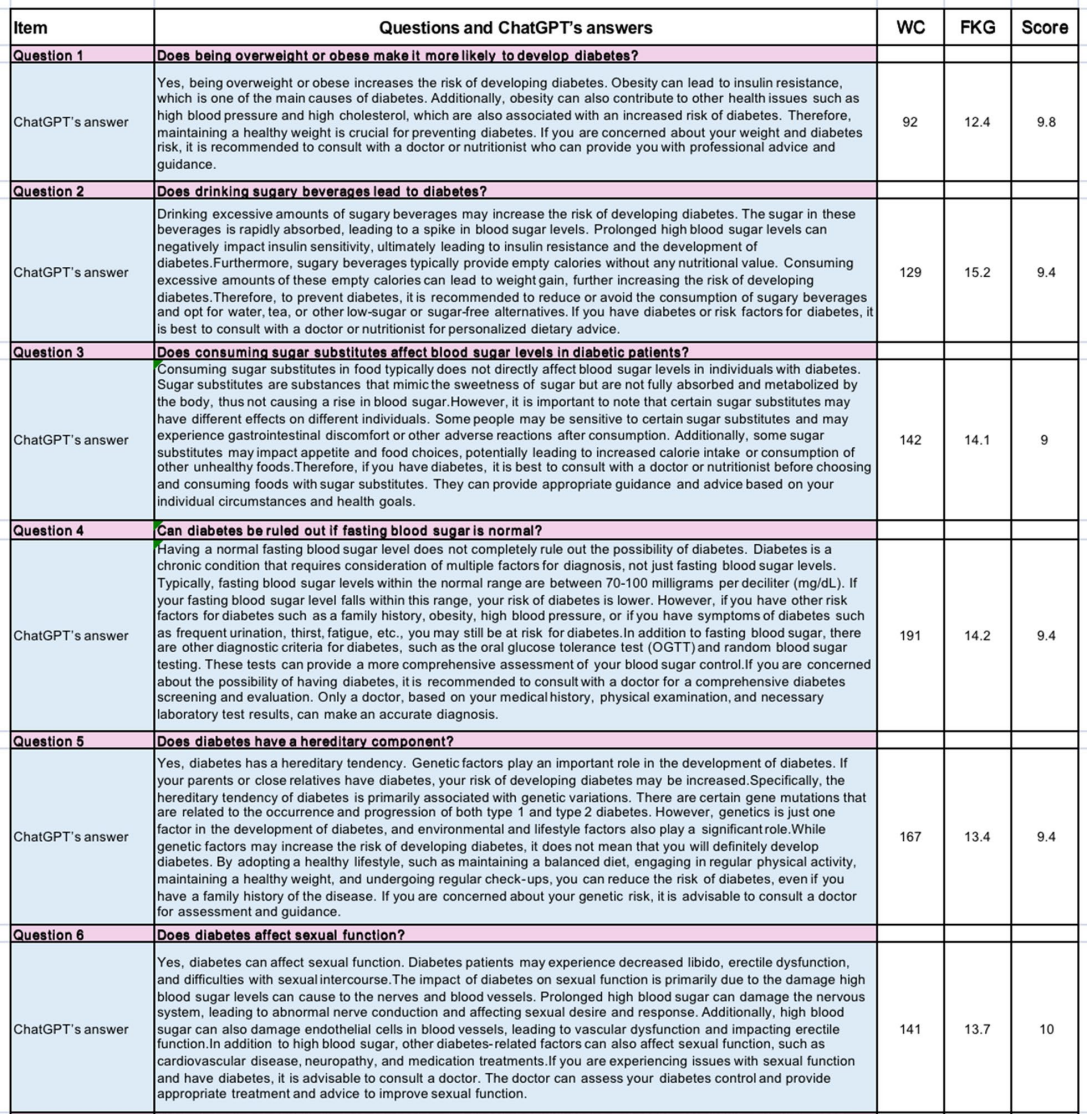

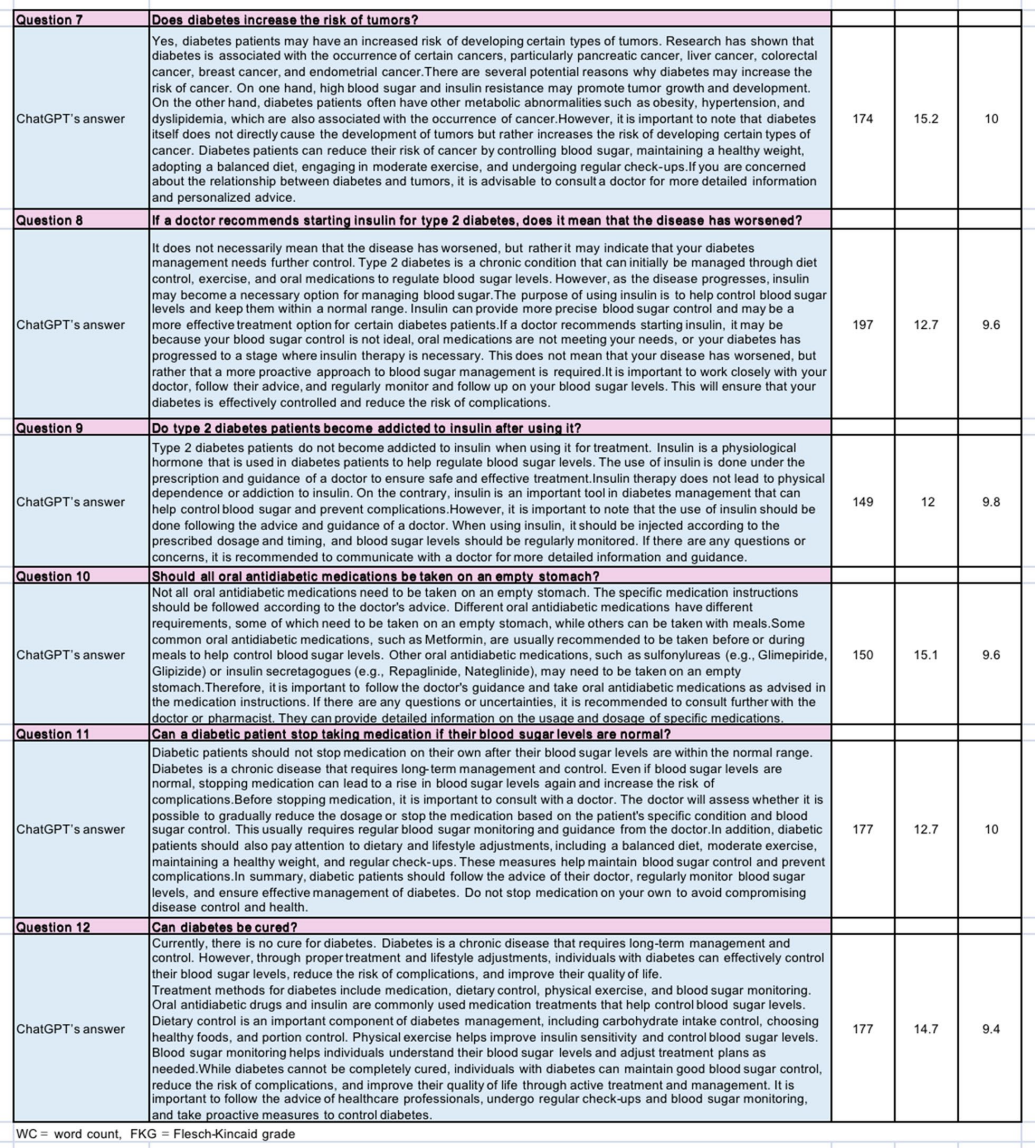
Diabetes knowledge misconceptions and ChatGPT’s answers. **A** Q1–Q6 and ChatGPT’s answers. **B** Q7–Q12 and ChatGPT’s answers

In this research, we employed web searches to identify prevalent inquiries and misunderstandings regarding diabetes-related knowledge. Frequently encountered questions were selected and presented to ChatGPT (ChatGPT-3.5-turbo, mode), with the generated responses being recorded (Fig. 1A, B). The quality of these responses was independently evaluated by experienced professionals in the field of endocrinology. Evaluation scores ranged from 0 to 10, with 10 indicating high accuracy, 8 ≤ score < 10 representing fairly accurate responses, 6 ≤ score < 8 indicating average accuracy, and scores below 6 indicating inaccuracies. From July 1, 2023, to July 3, 2023, five experts assessed and analyzed the limitations of ChatGPT's answers.

## Results

**Fig. 2:**
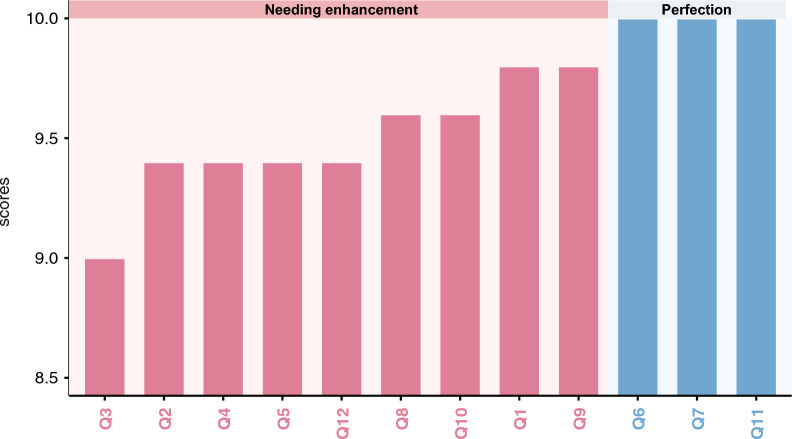
Evaluation ChatGPT’s answers’ scores and suggestions

Each response provided by ChatGPT consists of approximately 157 ± 29 words, and the Flesch-Kincaid Grade Level averages at 13.8 ± 1.1. Out of the 12 answers evaluated, 3 received a rating of 10, indicating a high level of accuracy. The remaining 9 answers had an average ± standard deviation rating of 9.5 ± 0.2, indicating a consistently high level of accuracy (Fig. 2). To examine the impact of repeated questions on the output answers, we conducted five runs for each of the 12 questions. The results revealed slight variations in sentence structure, but the answers remained consistent.

The findings indicate that ChatGPT generally provides reasonably accurate information regarding misconceptions about diabetes. However, experts have identified certain instances where ChatGPT's responses lack completeness and precision. For instance, in response to question 3: "Does consuming sugar substitutes affect blood sugar levels in diabetic patients?", ChatGPT's answer stating that it does not cause blood sugar elevation is not sufficiently accurate. Research conducted by Mathur et al. [3] suggests that the use of sugar substitutes can increase insulin resistance. Similarly, for question 4: "Can diabetes be ruled out if fasting blood sugar is normal?", ChatGPT's response stating that the normal range for fasting blood sugar is 70–100 mg per deciliter (mg/dL) is incorrect. The current standard for fasting blood sugar has been adjusted to 79–110 mg/dL. Additionally, in response to question 12: "Can diabetes be cured?", ChatGPT's answer stating that it cannot be cured is not entirely accurate. Research indicates that surgical treatment can lead to remission of diabetes in obese patients [4].

ChatGPT’s answers may lack completeness and precision due to its reliance on existing information and text, without real-time updating capabilities. As ChatGPT is not connected to the internet and has limited knowledge, there is a possibility of generating inaccurate or biased content. However, users have the option to provide feedback using the "Not Satisfied" button, enabling ChatGPT to learn and enhance its responses.

## Limitations

This study encountered several limitations. Firstly, we did not evaluate patients’ ratings of ChatGPT’s answers, and their empathetic assessments may vary from those of healthcare professionals. Secondly, ChatGPT does not provide citations, preventing users from verifying the accuracy of the information or delving deeper into the topic. Thirdly, although we assessed a range of questions, the selected ones may not encompass all the diabetes-related issues comprehensively.

## Conclusion

Our study revealed that ChatGPT has the potential to offer valuable and precise health information regarding diabetes. Nonetheless, further investigation is necessary to ascertain the consistent accuracy of artificial intelligence in providing diabetes-related information. Additionally, it is crucial to establish supervisory mechanisms to evaluate the quality of information delivered by chatbots and other AI systems. Furthermore, real-time updates of health information are essential to cater to the requirements of individuals with diabetes.

## Data Availability

Not applicable.
